# Circadian Blood Pressure Pattern and Microvascular and Macrovascular Cerebral Imaging Burden

**DOI:** 10.3390/jcm15135038

**Published:** 2026-06-28

**Authors:** Enise Nur Özlem Tiryaki, Uğur Karagöz, Muhammet Mücahit Tiryaki

**Affiliations:** 1 Department of Neurology, Yalova Training and Research Hospital, Yalova 77200, Türkiye; 2 Department of Cardiology, Izmir Katip Celebi University, Ataturk Training and Research Hospital, Izmir 35620, Türkiye; ugur.karagoz@ikc.edu.tr; 3 Department of Cardiology, Faculty of Medicine, Yalova University, Yalova 77200, Türkiye; muhammet.tiryaki@yalova.edu.tr

**Keywords:** non-dipping, reverse dipping, white matter hyperintensities, intracranial arterial calcification, ambulatory blood pressure monitoring, Fazekas scale

## Abstract

**Background:** Nocturnal blood pressure (BP) dipping patterns have been associated with cerebrovascular disease, but the differential impact of non-dipping versus reverse dipping on white matter hyperintensity (WMH) burden and intracranial arterial calcification (IAC) remains unclear. **Methods**: This retrospective study included 376 patients who underwent 24 h ambulatory BP monitoring, brain MRI, and cranial CT. Patients were classified as dipper (≥10% SBP decline, n = 148), non-dipper (0–10%, n = 156), or reverse dipper (<0%, n = 72). WMH was assessed using the Fazekas scale (0–3) and analyzed with ordinal logistic regression. IAC was scored as present/absent. Multivariable models adjusted for age, sex, hypertension, diabetes, chronic kidney disease (CKD), and coronary artery disease (CAD). **Results**: Reverse dippers had significantly higher Fazekas grades (OR 2.26; 95% CI 1.13–4.56; *p* = 0.022) and IAC prevalence (OR 8.03; 95% CI 2.42–29.18; *p* = 0.001) compared to dippers. Non-dippers showed no association with Fazekas (OR 0.78; 0.45–1.36; *p* = 0.386) but were independently associated with IAC (OR 3.75; 1.43–10.76; *p* = 0.010). A significant dose–response trend was observed for IAC (OR 2.89 per category; 1.60–5.47; *p* = 0.001) but not for Fazekas grade (OR 1.35; 0.97–1.90; *p* = 0.078). Age, diabetes, and CAD were also independent predictors of IAC, while female sex was protective. **Conclusions**: Reverse dipping was associated with both higher WMH burden and IAC, whereas non-dipping was associated mainly with IAC. These findings suggest that nocturnal BP phenotypes may be differentially associated with cerebral microvascular and macrovascular imaging markers. Further prospective studies are needed to confirm these observations.

## 1. Introduction

Hypertension is one of the most prevalent and modifiable risk factors for cerebrovascular disease, contributing to both small-vessel and large-vessel pathology through sustained hemodynamic stress, endothelial dysfunction, and accelerated arterial remodeling [1,2,3]. In the central nervous system, the structural consequences of chronic hypertension span a broad spectrum of neuroimaging phenotypes. White matter hyperintensities (WMH), detectable on brain magnetic resonance imaging, are well-established markers of cerebral small vessel disease and are independently associated with increased risks of stroke, cognitive impairment, and functional decline [4,5,6]. Intracranial arterial calcification (IAC), identifiable on non-contrast cranial computed tomography, has emerged as a marker of macrovascular aging and intracranial atherosclerotic burden, and has been associated with ischemic stroke and cognitive dysfunction [7,8,9]. Although WMH and IAC represent pathologically distinct neuroimaging entities, accumulating evidence suggests they may share overlapping mechanisms involving chronic hemodynamic stress, endothelial injury, and arterial wall remodeling, and their co-occurrence may reflect a broader, combined cerebrovascular phenotype [9,10].

Beyond absolute blood pressure levels, the circadian pattern of blood pressure regulation has emerged as an important marker of cardiovascular and cerebrovascular risk that is not fully captured by office or mean 24 h measurements [11]. Under physiological conditions, blood pressure declines by at least 10% during sleep, a phenomenon termed dipping. Ambulatory blood pressure monitoring (ABPM) allows quantification of this nocturnal fall and enables classification into three distinct phenotypes: dippers (nocturnal systolic blood pressure decline ≥10%), non-dippers (0 to <10%), and reverse dippers (<0%, i.e., nocturnal blood pressure exceeding daytime values) [12]. This three-category classification reflects a continuum of increasing nocturnal hemodynamic burden. Both non-dipping and reverse dipping have been associated with adverse cardiovascular outcomes and target organ damage, with reverse dipping often representing the most adverse phenotype [13,14]. The pathophysiological basis of non-dipping involves perturbations of autonomic regulation, circadian clock function, and renal sodium handling, processes that are likely amplified in reverse dippers, who exhibit true nocturnal hypertension rather than merely a blunted nocturnal decline [15].

Despite this evidence, important gaps remain in the neuroimaging literature. Abnormal nocturnal blood pressure regulation may promote cerebrovascular injury through impaired cerebral autoregulation and sustained hemodynamic stress affecting both small and large intracranial vessels [9,16]. Although the association between non-dipping BP patterns and cerebrovascular disease has been previously described, many available studies have used a binary dipper/non-dipper classification or pooled conventional non-dippers and reverse dippers into a single abnormal dipping category. A meta-analysis demonstrated an association between non-dipping status and silent cerebral small vessel disease including WMH, but the differential contribution of reverse dipping to cerebral microvascular injury remains incompletely defined [17]. This distinction may be clinically relevant because conventional non-dipping represents a blunted nocturnal BP decline, whereas reverse dipping reflects an actual nocturnal BP rise. In addition, the relationship between dipping phenotype and CT-detected IAC has received limited investigation [7,8,18]. To our knowledge, limited data exist on the simultaneous evaluation of dipper, non-dipper, and reverse dipper phenotypes with both WMH burden and IAC presence in the same cohort.

Accordingly, we investigated the differential associations of ABPM-derived dipping phenotypes with cerebral microvascular and macrovascular imaging markers, defined as WMH burden using the ordinal Fazekas scale and CT-detected IAC presence. We specifically aimed to determine whether reverse dipping shows a broader adverse neuroimaging profile than conventional non-dipping, after adjustment for major vascular risk factors.

## 2. Materials and Methods

### 2.1. Study Design and Population

This retrospective study included consecutive adult patients evaluated in neurology and cardiology outpatient clinics between January 2025 and December 2025. Patients were screened for eligibility if they had clinically indicated 24 h ABPM and underwent both brain MRI and non-contrast cranial CT within one month of ABPM. ABPM was performed as part of routine clinical evaluation for suspected or established hypertension, assessment of blood pressure control, suspected abnormal nocturnal blood pressure pattern, or symptoms potentially related to blood pressure variability. Brain MRI was obtained for clinical neurological indications, including headache, dizziness, migraine-like symptoms, syncope or presyncope, nonspecific neurological symptoms, or evaluation of possible cerebrovascular disease. Non-contrast cranial CT was performed as part of routine neurological assessment when clinically indicated, particularly for initial evaluation of neurological symptoms or cerebrovascular disease. Patients were eligible if aged ≥18 years. Patients were excluded if ABPM recordings were technically invalid or incomplete, if brain MRI or cranial CT was unavailable or of insufficient quality for assessment, or if they had central nervous system disorders that could substantially confound white matter hyperintensity evaluation, such as brain tumors, multiple sclerosis/demyelinating disease, or other major structural central nervous system lesions. The patient selection process is summarized in Supplementary Figure S1.

Demographic and clinical variables, including age, sex, hypertension, diabetes mellitus, chronic kidney disease, coronary artery disease, cerebrovascular disease, number of antihypertensive medications, and combined antihypertensive therapy, were obtained from the medical records. Headache and dizziness were recorded based on patient-reported complaints documented during routine clinical evaluation, whereas migraine status was determined according to a previously established diagnosis recorded in the medical records.

Ambulatory BP monitoring was performed using a validated oscillometric ABPM device (Microlife WatchBP O3; Microlife AG, Widnau, Switzerland). BP measurements were obtained every 30 min during the daytime (07:00–23:00) and every 60 min during the nighttime (23:00–07:00). ABPM recordings were considered valid if they had a successful reading rate of at least 80% and a total recording duration of at least 22 h. Recordings with insufficient valid measurements or incomplete day/night data were excluded from analysis. Mean 24 h, daytime, and nighttime systolic and diastolic BP values were obtained from the ABPM report. Nocturnal systolic BP decline was calculated as the percentage reduction in mean nighttime SBP relative to mean daytime SBP. Patients were classified into three groups: dippers (10–20% decline, including 4 extreme dippers with >20% decline, n = 148), non-dippers (0–10% decline, n = 156), and reverse dippers (<0% decline, indicating that mean nighttime SBP exceeded mean daytime SBP, n = 72). WMH burden was graded using a global Fazekas scale ranging from 0 to 3, where grade 0 indicated absent WMH, grade 1 punctate foci, grade 2 early confluent lesions, and grade 3 large confluent lesions [19]. Periventricular and deep WMH sub-scores were not analyzed separately because separate subregional scores were not systematically available in the retrospective dataset. Fazekas grade was analyzed as an ordinal variable using proportional odds logistic regression. IAC was assessed as a binary variable, defined as the presence or absence of visible intracranial arterial calcification on cranial CT. Quantitative calcification burden, arterial segment involvement, laterality, and severity grading were not systematically available in the retrospective dataset and therefore were not included in the analysis. Fazekas scoring and CT-based IAC assessment were performed independently by two experienced clinicians who were blinded to the ABPM-derived dipping classification and to each other’s evaluations. Inter-rater agreement was evaluated using weighted Cohen’s kappa for Fazekas grade and Cohen’s kappa for binary IAC assessment. Disagreements were resolved by consensus, and the consensus scores were used for the final analysis. The main imaging outcomes of interest were Fazekas burden and CT-detected intracranial arterial calcification, whereas symptom variables were evaluated only in supplementary analyses. The study protocol was approved by the local ethics committee, which waived the requirement for individual written informed consent given the retrospective design and the use of anonymized, routinely collected clinical data. The study was conducted in accordance with the principles of the Declaration of Helsinki. (0244-12/03/2026).

### 2.2. Statistical Analysis

Statistical analyses were performed using R version 4.4.0 (R Foundation for Statistical Computing, Vienna, Austria). Continuous variables are presented as median and interquartile range (IQR), and categorical variables as number and percentage. Between-group comparisons used Kruskal–Wallis or Fisher’s exact test, as appropriate. Ordinal logistic regression (proportional odds model) was used to assess associations with Fazekas grade (0–3), reporting common odds ratios (OR). The proportional odds assumption for the ordinal logistic regression model was assessed using nominal and scale tests. Because the assumption was not fully supported for all covariates, an additional sensitivity analysis was performed using high Fazekas burden as a binary outcome, defined as Fazekas grades 2–3 versus grades 0–1. Binary logistic regression was used for IAC presence. Covariates included dipping status (reference: dipper), age, sex, hypertension, diabetes, CKD, and (for IAC) CAD. Trend analysis was performed by treating dipping categories as an ordinal score (0 = dipper, 1 = non-dipper, 2 = reverse dipper). To address potential confounding by absolute BP burden and antihypertensive treatment intensity, additional sensitivity models were constructed by separately adding 24 h mean systolic BP, daytime mean systolic BP, nighttime mean systolic BP, or 24 h pulse pressure together with the number of antihypertensive medications to the multivariable models. These ABPM parameters were not entered simultaneously because of potential collinearity among ambulatory BP measures. Nocturnal systolic BP decline was also analyzed as a continuous variable. Results are reported as odds ratios (ORs) with 95% confidence intervals (CIs). All tests were two-sided, and a *p*-value <0.05 was considered statistically significant.

## 3. Results

A total of 376 patients were included (148 dippers, 156 non-dippers, 72 reverse dippers). Baseline characteristics are shown in Table 1. Reverse dippers were significantly older (median 60 years), had higher prevalence of hypertension (67%) and CKD (17%), and used more antihypertensive drugs (*p* = 0.020) compared to dippers and non-dippers. Non-dippers and dippers had similar age (45 vs. 46 years) and similar 24 h BP levels. Notably, reverse dippers had higher 24 h pulse pressure (*p* = 0.009) and lower heart rate (*p* = 0.002).

Neuroimaging findings differed markedly: reverse dippers had the highest proportion of Fazekas grade 2–3 (44% vs. 11% in dippers and 15% in non-dippers, *p* < 0.001) and the highest IAC prevalence (50% vs. 14% in dippers and 23% in non-dippers, *p* < 0.001). Inter-rater agreement was high for both imaging outcomes, with weighted Cohen’s kappa of 0.82 for Fazekas scoring and Cohen’s kappa of 0.84 for IAC assessment (both *p* < 0.001). All discrepancies were resolved by consensus. In supplementary analyses (Supplementary Table S1), headache was most frequent in non-dippers and least frequent in reverse dippers compared with dippers (*p* < 0.001). Migraine prevalence was lowest in non-dippers (7.7%) and highest in reverse dippers (27.8%; *p* < 0.001). Dizziness was most frequent among reverse dippers (47.2%), compared with non-dippers (31.4%) and dippers (25.0%; *p* = 0.004).

Table 2 summarizes the multivariable models. For Fazekas grade (ordinal), reverse dipping remained independently associated with higher WMH burden (OR 2.26; 95% CI 1.13–4.56; *p* = 0.022), whereas non-dipping was not (OR 0.78; 0.45–1.36; *p* = 0.386). Age was strongly associated (OR 1.15 per year; *p* < 0.001), and female sex was protective (OR 0.33; *p* < 0.001). The trend across dipper, non-dipper, and reverse dipper was not significant (OR 1.35; 0.97–1.90; *p* = 0.078). For IAC, both non-dipping (OR 3.75; 1.43–10.76; *p* = 0.010) and reverse dipping (OR 8.03; 2.42–29.18; *p* = 0.001) were independently associated with IAC presence. Age (OR 1.17; *p* < 0.001), diabetes (OR 10.85; *p* = 0.008), and CAD (OR 11.60; *p* < 0.001) were also significant, while female sex was protective (OR 0.25; *p* = 0.002). A significant dose–response trend was observed across dipping categories (OR 2.89; 1.60–5.47; *p* = 0.001). The proportional odds assumption was formally assessed for the ordinal Fazekas model. Although no statistically significant violation was observed for the dipping phenotype, the assumption was not fully supported for all covariates. Therefore, a sensitivity analysis was performed using high Fazekas burden as a binary outcome. In this model, reverse dipping remained associated with high Fazekas burden (OR, 3.40; 95% CI, 1.37–8.67; *p* = 0.009), whereas non-dipping was not significantly associated with this outcome (OR, 1.41; 95% CI, 0.62–3.25; *p* = 0.409).

Adding hypertension duration to the fully adjusted models did not materially change any point estimates (reverse dipping OR for Fazekas changed from 2.26 to 2.25; for IAC from 8.03 to 7.28), and hypertension duration itself was not independently associated with either outcome (*p* > 0.7 in all models). Because hypertension duration was retrospectively recorded, this exploratory analysis should be interpreted cautiously. Additional sensitivity analyses were performed to assess whether the observed associations were explained by absolute ABPM parameters or antihypertensive medication burden. After additional adjustment for 24 h mean systolic BP, daytime mean systolic BP, or 24 h pulse pressure together with the number of antihypertensive medications, reverse dipping remained associated with higher Fazekas grade. However, this association was attenuated after adjustment for nighttime systolic BP. For IAC, reverse dipping remained associated with calcification after adjustment for nighttime systolic BP, although the effect size was reduced. When nocturnal systolic BP decline was analyzed as a continuous variable, each 1% greater nocturnal decline was associated with lower odds of higher Fazekas grade (OR, 0.95; 95% CI, 0.92–0.99) and IAC presence (OR, 0.87; 95% CI, 0.81–0.93). The full results of these sensitivity analyses are provided in Supplementary Table S2.

**Figure 1:**
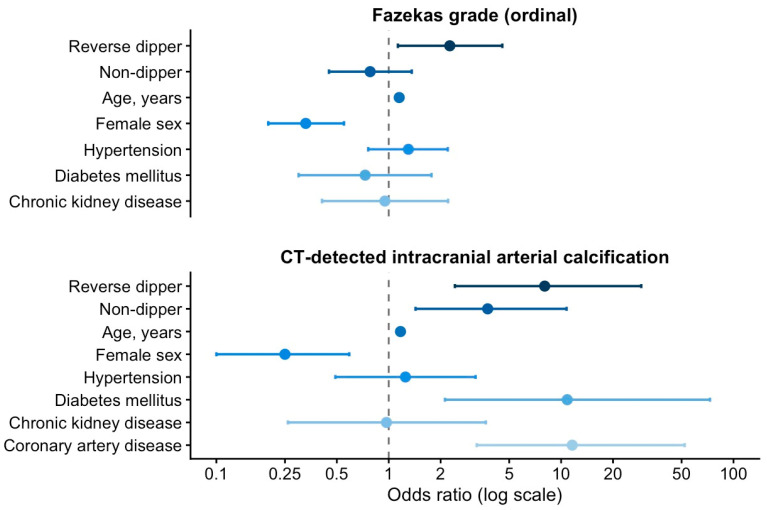
Forest plot showing multivariable-adjusted odds ratios for Fazekas grade and CT-detected intracranial arterial calcification according to nocturnal blood pressure dipping status and clinical covariates. Horizontal lines indicate 95% confidence interval.

The multivariable-adjusted associations are also illustrated in Figure 1.

## 4. Discussion

The present study suggests that ABPM-derived nocturnal BP phenotypes are differentially associated with cerebrovascular imaging markers. Reverse dipping was associated with both higher WMH burden and greater IAC prevalence, whereas conventional non-dipping was associated mainly with IAC. These observations were made in a cohort in which mean 24 h BP values were broadly comparable across dipping groups, supporting the concept that the circadian distribution of BP may provide information not fully captured by average ambulatory BP values. However, additional ABPM-adjusted analyses refined this interpretation: the association between reverse dipping and WMH burden was attenuated after adjustment for nighttime systolic BP, whereas the association with IAC persisted, although with a reduced effect size. Therefore, reverse dipping may be interpreted as a clinically recognizable phenotype of nocturnal hemodynamic dysregulation linked to cerebrovascular imaging burden, rather than as an entirely BP-independent determinant.

The association of reverse dipping with higher WMH burden is biologically plausible and consistent with the known vulnerability of the cerebral microvasculature to sustained hemodynamic stress. WMH are thought to arise from chronic ischemic injury to the periventricular and subcortical white matter, mediated by endothelial dysfunction, impaired cerebral autoregulation, and blood–brain barrier disruption in the deep penetrating arterioles that supply these territories [16,20]. An important distinction between non-dipping and reverse dipping is that reverse dippers experience not merely insufficient nocturnal blood pressure reduction but true nocturnal hypertension, with blood pressure during sleep exceeding daytime values [21]. This sustained hemodynamic load during the physiological recovery period may generate a degree of cumulative microvascular stress. The pathophysiological substrate of non-dipping encompasses perturbations of autonomic tone, circadian clock regulation, and nocturnal sodium excretion, and these disturbances are likely amplified in the reverse dipping phenotype, potentially augmenting endothelial injury and impairing vascular repair mechanisms during sleep [15]. The absence of a significant WMH association in non-dippers in the present cohort is broadly consistent with the prior literature when interpreted in context. A meta-analysis of 14 studies demonstrated an association between non-dipping and silent cerebral small vessel disease including WMH but noted substantial heterogeneity across studies [17]. Many of those studies used a binary dipper/non-dipper classification or pooled non-dippers and reverse dippers into a single category. If the WMH signal is driven predominantly by the reverse dipping subgroup, as the present data suggest, pooling these two phenotypes would be expected to produce attenuated and heterogeneous associations, consistent with what was observed in that meta-analysis. In this sense, the null finding for non-dipping may reflect the importance of distinguishing the two phenotypes rather than indicating a true absence of biological relevance in the non-dipper group per se. In our sensitivity analyses, the association between reverse dipping and Fazekas burden persisted after adjustment for 24 h mean systolic BP, daytime systolic BP, and pulse pressure, but was attenuated after adjustment for nighttime systolic BP. This pattern suggests that nighttime systolic BP load may be an important explanatory pathway linking reverse dipping to WMH burden.

Beyond microvascular pathology, the present study also observed an association between non-dipping status and intracranial arterial calcification. IAC reflects calcium deposition within the intimal or medial layer of intracranial arteries, a process driven by chronic hemodynamic stress, vascular inflammation, oxidative injury, and progressive arterial stiffness [8,18]. IAC is increasingly considered an imaging marker of intracranial atherosclerotic burden and cumulative vascular injury [16,22]. Even a blunted but still-positive nocturnal blood pressure fall may sustain sufficient cumulative pulsatile hemodynamic exposure on larger intracranial arteries to promote arterial wall remodeling and calcification, through a pathway that likely operates at a lower intensity threshold than the microvascular mechanisms underlying WMH. These differential threshold effects across vascular compartments are consistent with evidence that macrovascular and microvascular manifestations of hypertensive cerebrovascular disease can have distinct pathophysiological determinants [4,9]. In sensitivity analyses, the association between conventional non-dipping and IAC was attenuated after adjustment for nighttime systolic BP, suggesting that the relationship between abnormal dipping patterns and IAC may also be partly related to nocturnal BP load.

The dose–response relationship observed for IAC across the three dipping categories indicates that the risk of intracranial arterial calcification increases incrementally with the severity of nocturnal blood pressure dysregulation, from physiological dipping through non-dipping to true nocturnal hypertension in reverse dippers. This pattern parallels the dose–response relationship between dipping phenotype severity and adverse cardiovascular outcomes reported in large prospective cohorts and collaborative meta-analyses, and supports the concept of a continuum of intracranial vascular injury driven by escalating nocturnal hemodynamic burden [13,14]. Studies in non-dipping populations have documented augmented endothelial dysfunction, increased oxidative stress, and elevated proinflammatory adhesion molecule expression compared with normal dippers, and these mechanisms are likely further amplified in reverse dippers, who sustain higher blood pressure levels throughout the entire sleep period [22,23]. The IAC findings appeared more robust across ABPM-adjusted models than the WMH findings. Reverse dipping remained associated with IAC even after adjustment for nighttime systolic BP, although the effect size was attenuated. This pattern suggests that IAC may reflect a cumulative macrovascular phenotype related to both nocturnal BP load and broader systemic vascular risk.

The present findings are further supported by prior neuroimaging evidence. Nighttime blood pressure characteristics have been linked to subclinical cerebrovascular injury in earlier studies [24]. Regarding IAC, the dose–response pattern across dipping categories is consistent with evidence linking intracranial arterial calcification to systemic atherosclerotic burden and cumulative vascular injury, and with reports of a correlation between IAC severity and overall cerebral small vessel disease burden [7,9,10,18,25]. The continuous analysis of nocturnal systolic BP decline further supports a graded relationship between circadian BP regulation and cerebrovascular imaging burden. Each 1% greater nocturnal systolic BP decline was associated with lower odds of both higher Fazekas grade and IAC presence. This finding complements the categorical dipping analysis and suggests that the relationship between nocturnal BP regulation and cerebrovascular imaging markers is not restricted to predefined dipping categories.

The independent associations of older age with both WMH burden and IAC are consistent with the established contribution of cumulative vascular aging to both microvascular rarefaction and intracranial arterial remodeling [26,27]. The strong and independent associations of diabetes mellitus and coronary artery disease with IAC, but not with WMH burden, support the interpretation that CT-detected IAC reflects a systemic atherosclerotic phenotype rather than an isolated intracranial finding, and reinforce its potential value as a marker of global vascular burden on cranial CT [18,25]. Female sex was inversely associated with both imaging outcomes after multivariable adjustment, likely reflecting sex-related differences in arterial stiffness and atherosclerotic burden; this observation warrants cautious interpretation in the context of the cross-sectional design. Notably, a diagnosis of hypertension was not independently associated with either outcome after multivariable adjustment, suggesting that ABPM-derived nocturnal BP characteristics may provide additional information beyond hypertension status alone in this cohort. In exploratory analyses, dizziness was more frequently reported among reverse dippers, while headache and migraine frequencies differed across groups; given the heterogeneous etiology of dizziness and the retrospective nature of symptom recording, these findings should not be interpreted causally, but may suggest that altered circadian hemodynamics carry clinical correlates that extend beyond structural imaging changes.

This study has several limitations. First, its retrospective, single-center design precludes causal inference and may limit generalizability to community-based or asymptomatic populations. Second, IAC was assessed as a binary variable; quantitative calcification burden, arterial segment involvement, laterality, and severity were not available. Therefore, the findings should be interpreted as associations with CT-detected IAC presence rather than with the extent or anatomical distribution of calcification. Similarly, WMH burden was evaluated using the Fazekas scale rather than volumetric methods. Third, the small number of patients with Fazekas grade 3 and the wide confidence intervals in the IAC models indicate statistical imprecision; therefore, the magnitude of the observed associations should be interpreted cautiously. Fourth, the dipping groups were not fully balanced, and reverse dippers were older and had a higher vascular risk burden. Although multivariable and ABPM-adjusted sensitivity analyses were performed, residual confounding cannot be excluded. Fifth, reverse dipping and elevated nighttime BP load are closely related, and this study cannot fully disentangle whether reverse dipping represents a distinct phenotype or primarily reflects nocturnal hypertension. Finally, sleep-related factors that may influence nocturnal BP patterns, including sleep quality, obstructive sleep apnea, nocturnal awakenings, sedative or hypnotic medication use, and shift-work status, were not systematically recorded.

## 5. Conclusions

In conclusion, reverse dipping was associated with both higher WMH burden and IAC, whereas non-dipping was associated mainly with IAC in this retrospective cohort. Additional analyses suggested that the association between reverse dipping and WMH may be partly explained by nighttime systolic BP load, while the association with IAC persisted across ABPM-adjusted models. These findings suggest that nocturnal BP phenotypes, particularly reverse dipping, may be differentially linked to cerebral microvascular and macrovascular imaging markers. Prospective studies using quantitative imaging and longitudinal ABPM assessment are needed to confirm these observations.

## Figures and Tables

**Table 1:** Baseline clinical, ambulatory blood pressure monitoring, and neuroimaging characteristics according to nocturnal blood pressure dipping status.

Variables	Overall (N = 376)	Dipper (n = 148)	Non-Dipper (n = 156)	Reverse Dipper (n = 72)	*p*-Value
Age, years	47.5 (36.0–62.0)	46 (36–56)	45 (36–59)	60 (46–73)	<0.001
Female sex, n (%)	232 (61.7%)	88 (59)	100 (64)	44 (61)	0.704
Hypertension, n (%)	176 (46.8%)	68 (46)	60 (38)	48 (67)	<0.001
Diabetes mellitus, n (%)	20 (5.3%)	8 (5)	8 (5)	4 (6)	0.997
Chronic kidney disease, n (%)	24 (6.4%)	4 (3)	8 (5)	12 (17)	<0.001
Coronary artery disease, n (%)	32 (8.5%)	12 (8)	12 (8)	8 (11)	0.705
Cerebrovascular disease, n (%)	8 (2.1%)	4 (2.7%)	4 (1.8%)	0 (0)	0.717
Number of antihypertensive drugs, n (%)					0.020
0	212 (56.4%)	92 (62.2)	88 (56.4)	32 (44.4)	
1	76 (20.2%)	24 (16.2)	36 (23.1)	16 (22.2)	
2	56 (14.9%)	20 (13.5)	16 (10.3)	20 (27.8)	
3	32 (8.5%)	12 (8.1)	16 (10.3)	4 (5.6)	
Combined antihypertensive therapy, n (%)	88 (23.4%)	32 (21.6)	32 (20.5)	24 (33.3)	0.091
24 h mean systolic BP, mmHg	117.5 (106.0–127.0)	120 (106–127)	116 (107–124)	120 (104–138)	0.088
24 h mean diastolic BP, mmHg	71.0 (65.0–77.0)	73 (68–78)	70 (65–77)	72 (63–81)	0.204
24 h mean heart rate, bpm	75.0 (70.0–81.0)	77 (71–82)	75 (70–80)	73 (68–77)	0.002
24 h pulse pressure, mmHg	44.0 (40.0–50.0)	44 (40–50)	43 (39–49)	46 (41–57)	0.009
Nocturnal SBP decline, %	7.2 (2.0–12.8)	13.8 (12.0–15.4)	4.6 (3.1–7.4)	−4.2 (−8.2–−1)	<0.001
Fazekas score, n (%)					<0.001
0	216 (57.4)	88 (59)	100 (64)	28 (39)	
1	88 (23.4)	44 (30)	32 (21)	12 (17)	
2	68 (18.1)	16 (11)	24 (15)	28 (39)	
3	4 (1)	0 (0)	0 (0)	4 (6)	
CT-detected intracranial arterial calcification, n (%)	92 (24.5%)	20 (14)	36 (23)	36 (50)	<0.001
Hypertension duration, years	7 (6–7)	6 (6–7)	7 (6–8)	7 (7–8)	<0.001

Data are presented as median (IQR) or n (%), as appropriate. *p*-values were calculated using the Kruskal–Wallis test for continuous variables and Fisher’s exact test for categorical variables. Hypertension duration is reported only among patients with a documented diagnosis of hypertension. BP, blood pressure; bpm, beats per minute; CT, computed tomography; h, hour.

**Table 2:** Multivariable logistic regression models for Fazekas grade (ordinal) and CT-detected intracranial arterial calcification.

	Fazekas Grade (0–3, Ordinal)		CT-Detected Intracranial Arterial Calcification	
Variables	OR (95% CI)	*p*-Value	OR (95% CI)	*p*-Value
Non-dipper vs. dipper	0.78 (0.45–1.36)	0.386	3.75 (1.43–10.76)	0.010
Reverse dipper vs. dipper	2.26 (1.13–4.56)	0.022	8.03 (2.42–29.18)	0.001
Trend (per category)	1.35 (0.97–1.90)	0.078	2.89 (1.60–5.47)	0.001
Age, years	1.15 (1.12–1.18)	<0.001	1.17 (1.12–1.22)	<0.001
Female sex	0.33 (0.20–0.55)	<0.001	0.25 (0.10–0.59)	0.002
Hypertension	1.30 (0.76–2.20)	0.342	1.25 (0.49–3.19)	0.643
Diabetes mellitus	0.73 (0.30–1.77)	0.480	10.85 (2.12–73.05)	0.008
Chronic kidney disease	0.95 (0.41–2.21)	0.904	0.97 (0.26–3.66)	0.961
Coronary artery disease			11.60 (3.24–52.09)	<0.001

Values are presented as odds ratios with 95% confidence intervals. Fazekas grade was modeled using ordinal logistic regression and adjusted for dipping status, age, sex, hypertension, diabetes mellitus, and chronic kidney disease. IAC presence was modeled using binary logistic regression and adjusted for dipping status, age, sex, hypertension, diabetes mellitus, chronic kidney disease, and coronary artery disease. Trend was modeled as an ordinal score: 0 = dipper, 1 = non-dipper, 2 = reverse dipper. CI, confidence interval; IAC, intracranial arterial calcification; OR, odds ratio.

## Data Availability

The raw data supporting the conclusion of this article will be made available by the authors upon reasonable request.

## Supplementary Materials

The following supporting information can be downloaded at: https://www.mdpi.com/article/10.3390/jcm15135038/s1, Figure S1: Flow diagram of patient selection; Table S1: Symptom profile according to nocturnal blood pressure dipping status; Table S2: Sensitivity analyses adjusted for absolute ABPM parameters and antihypertensive medication burden.

## Abbreviations

ABPM	ambulatory blood pressure monitoring
BP	blood pressure
CAD	coronary artery disease
CI	confidence interval
CKD	chronic kidney disease
CT	computed tomography
DBP	diastolic blood pressure
IAC	intracranial arterial calcification
IQR	interquartile range
MRI	magnetic resonance imaging
OR	odds ratio
SBP	systolic blood pressure
WMH	white matter hyperintensities
