# Supplementary material for: Circadian Blood Pressure Pattern and Microvascular and Macrovascular Cerebral Imaging Burden
Source: J Clin Med. 2026 Jun 28;15(13):5038. doi: 10.3390/jcm15135038 (PMC13363054; doi:10.3390/jcm15135038)
Supplement: Supplementary file 1 [file jcm-15-05038-s001.zip › Supplementary files.pdf]

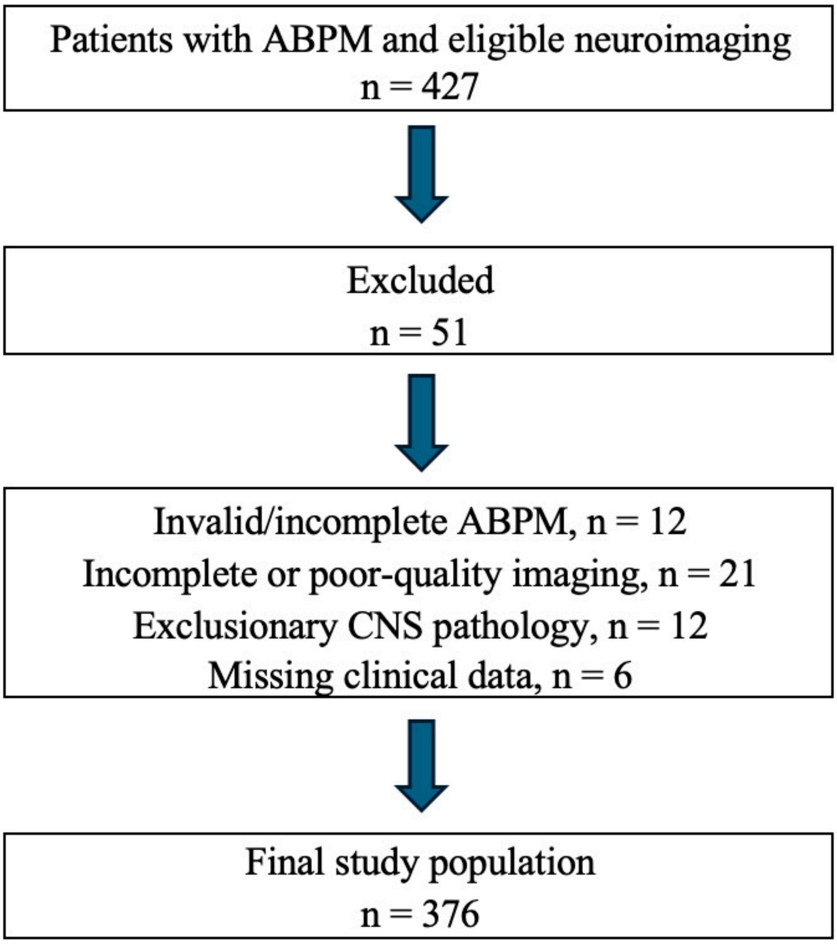

Supplementary Figure S1. Flow diagram of patient selection. ABPM, ambulatory blood pressure monitoring; CNS, central nervous system.

Supplementary Table S1. Symptom profile according to nocturnal blood pressure dipping status

Variables	Overall (N=376)	Dipper (n=148)	Non-dipper (n=156)	Reverse dipper (n=72)	p-value
**Headache**	232 (61.7%)	92 (62.2)	112 (71.8)	28 (38.9)	<0.001
**Migraine**	60 (16)	28 (18.9)	12 (7.7)	20 (27.8)	<0.001
**Dizziness**	120 (31.9)	37 (25)	49 (31.4)	34 (47.2)	0.004

Data are presented as n (%). P values were calculated using Fisher's exact test.

Supplementary Table S2. Sensitivity analyses adjusted for absolute ABPM parameters, antihypertensive medication burden, and continuous nocturnal SBP decline.

**Panel A.** Models additionally adjusted for absolute ABPM parameters and antihypertensive medication burden			
**Outcome**	**Additional adjustment**	**Non-dipper vs dipper OR (95% CI), p**	**Reverse dipper vs dipper OR (95% CI), p**
Fazekas grade	24-hour mean SBP + antihypertensive drug count	0.83 (0.47–1.47), p=0.532	2.48 (1.23–5.06), p=0.012
Fazekas grade	Daytime mean SBP + antihypertensive drug count	0.93 (0.52–1.67), p=0.815	3.11 (1.50–6.52), p=0.002
Fazekas grade	Nighttime mean SBP + antihypertensive drug count	0.66 (0.37–1.17), p=0.157	1.55 (0.74–3.27), p=0.244
Fazekas grade	24-hour pulse pressure + antihypertensive drug count	0.75 (0.43–1.31), p=0.318	2.31 (1.15–4.67), p=0.019
IAC presence	24-hour mean SBP + antihypertensive drug count	4.28 (1.54–13.2), p=0.007	11.7 (3.01–51.3), p<0.001
IAC presence	Daytime mean SBP + antihypertensive drug count	5.54 (1.95–17.9), p=0.002	18.1 (4.37–88.7), p<0.001
IAC presence	Nighttime mean SBP + antihypertensive drug count	2.39 (0.83–7.38), p=0.113	4.19 (1.07–17.0), p=0.041
IAC presence	24-hour pulse pressure + antihypertensive drug count	4.65 (1.58–15.8), p=0.008	14.6 (3.34–77.0), p<0.001
**Panel B.** Nocturnal SBP decline analyzed as a continuous variable			
**Outcome**	**Analysis**	**OR (95% CI)**	**p-value**
Fazekas grade	Nocturnal SBP decline, per 1% greater decline	0.95 (0.92–0.99)	0.006
IAC presence	Nocturnal SBP decline, per 1% greater decline	0.87 (0.81–0.93)	<0.001

Values are presented as odds ratios with 95% confidence intervals and p values. In Panel A, Fazekas grade was modeled using ordinal logistic regression, and IAC presence was modeled using binary logistic regression. Each model included dipping phenotype, the listed ABPM parameter, number of antihypertensive medications, and the covariates used in the primary multivariable model. ABPM parameters were entered separately because of potential collinearity among ambulatory BP measures. In Panel B, nocturnal SBP decline was analyzed as a continuous variable, with odds ratios expressed per 1% greater nocturnal decline. ABPM, ambulatory blood pressure monitoring; BP, blood pressure; CI, confidence interval; IAC, intracranial arterial calcification; OR, odds ratio; SBP, systolic blood pressure.
